# Secretory leukocyte protease inhibitor as a novel predictive biomarker in patients with diabetic kidney disease

**DOI:** 10.3389/fendo.2024.1334418

**Published:** 2024-03-04

**Authors:** Weiwei Sun, Hanwen Yang, Jiale Zhang, Shuwu Wei, Qiaoru Wu, Jie Yang, Can Cao, Zhaoli Cui, Huijuan Zheng, Yaoxian Wang

**Affiliations:** ^1^ Department of Nephrology and Endocrinology, Dongzhimen Hospital, Affiliated to Beijing University of Chinese Medicine, Beijing, China; ^2^ Renal Research Institution of Beijing University of Chinese Medicine, Beijing, China; ^3^ Department of Proctology, China-Japan Friendship Hospital, Beijing, China; ^4^ Key Laboratory of Chinese Internal Medicine of Ministry of Education and Beijing, Dongzhimen Hospital Affiliated to Beijing University of Chinese Medicine, Beijing, China

**Keywords:** diabetic kidney disease, secretory leucocyte protease inhibitor, prognosis, renal clinical endpoint events, biomarkers

## Abstract

**Background:**

Secretory leukocyte protease inhibitor (SLPI) is a multifunctional protein involved in the chronic inflammatory process, implicated in the pathogenesis of diabetic kidney disease (DKD). However, its potential as a diagnostic and prognostic biomarker of DKD has yet to be evaluated. This study explored the clinical utility of SLPI in the diagnosis and prognosis of renal endpoint events in patients with DKD.

**Methods:**

A multi-center cross-sectional study comprised of 266 patients with DKD and a predictive cohort study comprised of 120 patients with stage IV DKD conducted between December 2016 and January 2022. The clinical parameters were collected for statistical analysis, a multivariate Cox proportional hazards model was used to evaluate the independent risk factors for renal endpoints.

**Results:**

Serum SLPI levels gradually increased with DKD progression (p<0.01). A significant correlation was observed between serum SLPI levels and renal function in patients with DKD. The mean follow-up duration in this cohort study was 2.32 ± 1.30 years. Multivariate Cox regression analysis showed SLPI levels≥51.61ng/mL (HR=2.95, 95% CI[1.55, 5.60], p<0.01), 24h urinary protein levels≥3500 mg/24h (HR=3.02, 95% CI[1.66, 5.52], p<0.01), Alb levels<30g/l (HR=2.19, 95% CI[1.12, 4.28], p<0.05), HGB levels<13g/dl (HR=3.18, 95% CI[1.49, 6.80], p<0.01), and urea levels≥7.1 mmol/L (HR=8.27, 95% CI[1.96, 34.93], p<0.01) were the independent risk factors for renal endpoint events in DKD patients.

**Conclusions:**

Serum SLPI levels increased with DKD progression and were associated with clinical parameters of DKD. Moreover, elevated SLPI levels showed potential prognostic value for renal endpoint events in individuals with DKD. These findings validate the results of previous studies on SLPI in patients with DKD and provide new insights into the role of SLPI as a biomarker for the diagnosis and prognosis of DKD that require validation.

## 1 Introduction

The incidence of diabetic kidney disease (DKD) is increasing annually and has become the main cause of end-stage renal disease (ESRD) worldwide, posing a serious threat to the lives and health of patients (1). Numerous studies have indicated that the processes leading to DKD progression are heterogeneous and include inflammation, metabolic stress, and morphological kidney lesions, making it difficult to identify high-risk individuals who are more likely to develop DKD and ultimately progress to ESRD (2). In the past decade, several studies have reported associations between the occurrence and progression of DKD and proinflammatory and profibrotic markers (3–5). However, biomarkers for predicting the onset and progression of kidney disease in diabetes patients remain inefficient. Hence, extensive efforts are underway to identify and confirm new diagnostic and prognostic biomarkers that can lead to a better understanding of DKD.

Secretory leukocyte protein inhibitor (SLPI), a non-glycosylated cationic protein produced by mucous membrane epithelial cells, neutrophils, and macrophages, controls the excess release of host-secreted serine proteases in response to injurious stimuli (6). In addition to its antiprotease activity, SLPI has anti-inflammatory, antimicrobial, antiviral, and immunomodulatory properties (7). This inhibitor has emerged as a protective agent regulating the inflammatory cascade (8). SLPI was first discovered and isolated from the secretions of patients with chronic obstructive pulmonary disease and cystic fibrosis in the 1970 (9). Subsequently, SLPI was found to be expressed on several mucosal surfaces, including those of the respiratory, digestive, and reproductive tracts (10–13). In 2001, Ohlsson et al. (14) first observed the distinct expression of SLPI mRNA and protein in distal renal tubular cells. In contrast, few studies exist on the correlation between SLPI levels and kidney disease, with the literature mainly focusing on acute kidney injury after surgery and the evaluation of the condition before and after renal transplantation (15, 16). Recently, circulating SLPI has been reported to correlate with metabolic and inflammatory parameters in metabolic diseases (17). Given the common inflammatory and metabolic mechanisms in DKD, we speculated that SLPI may play a crucial role in the initiation and progression of DKD.

Our previous study on serum biomarker concentrations in patients with DKD showed a significant correlation between SLPI and renal function at various stages of DKD (18). Therefore, we expanded the sample size of this cross-sectional study to investigate the performance of SLPI as a complementary diagnostic and prognostic biomarker of DKD.

## 2 Materials and methods

### 2.1 Cross-sectional study

#### 2.1.1 Patients

A total of 266 patients with DKD aged 18-75 years participated in this study. This multi-center cross-sectional study was conducted from December 2016 to January 2022 at six hospitals in Beijing: Dongzhimen Hospital, Beijing University of Chinese Medicine, Beijing Hospital of Integrated Traditional Chinese and Western Medicine, Beijing Hospital of Traditional Chinese Medicine, Xiyuan Hospital, China Academy of Chinese Medical Sciences, and the Guang’anmen Hospital, China Academy of Chinese Medical Sciences. In addition, 20 healthy individuals and 20 patients with diabetes t without a diagnosis of DKD were selected as controls. The inclusion criteria were as follows: (1) early stage, urinary microalbumin excretion rate (UAER) of 30-300 mg/24h or urinary albumin-to-creatinine ratio (UACR) of 30-300 mg/g; (2) established stage, UAER>300 mg/24h or UACR>300 mg/g, or 24h urinary total protein (24h UTP)>0.5g and eGFR≥60mL/min/1.73m^2^; (3)advanced stage, 30≤eGFR<60mL/min/1.73 m^2^. The demographic and clinical data were recorded. Subjects meeting any of the following criteria were excluded: (1) severe infection, moderate and severe anemia, electrolyte disturbance, and acute complications of diabetes occurring within four weeks; (2) patients with severe diseases of the heart, brain, liver, and hematopoietic system and those on glucocorticoid or immunosuppressants in the last three months before admission; (3) oliguria, anuria, serious pleural effusion or ascites, severe edema, or mental illness; (4) patients who received a kidney transplant or dialysis treatment; (5) patients who were pregnant or preparing for pregnancy or lactation; (6) participants in other interventional clinical trials; (7) participants who did not provide signed informed consent.

#### 2.1.2 Clinical and laboratory measurements

Serum samples were collected and immediately frozen at −80 °C. The routine clinical parameters of patients with DKD included age, sex, body mass index (BMI), systolic blood pressure (SBP), diastolic blood pressure (DBP), serum creatinine (Scr), estimated glomerular filtration rate (eGFR), urea, blood uric acid (UA), albumin (ALB), 24h UTP, low-density lipoprotein (LDL), serum cholesterol (CHO), and triglyceride (TG) levels. The SLPI levels were measured using a Luminex liquid suspension chip (cat. LXSAHM; R&D Systems), according to the manufacturer’s instructions(Wayen Biotechnologies).

#### 2.1.3 Statistical analysis

Statistical analyses were performed using SPSS statistics software 24 (SPSS, Inc.). Enumeration data were expressed as percentages, and measurement data were expressed as the mean ± standard deviation (SD). Data showing a skewed distribution were expressed as the median and interquartile range (IQR). Categorical data were assessed using the chi-squared test. Continuous variables were compared using an independent group t-test or one-way ANOVA for normally distributed data.

Otherwise, the Mann–Whitney U test or Kruskal-Wallis H test was used for skewed distributions. Correlations between SLPI and clinical characteristics were analyzed using Pearson’s correlation. The performance of the SLPI as a diagnostic biomarker was tested using receiver operating characteristic (ROC) curve analysis, and the Youden index was used to determine the cutoff value. The area under the ROC curve (AUC) was calculated, with AUC values between 0.7~0.9 indicating a degree of accuracy for diagnosis. Statistical significance was set at p< 0.05.

### 2.2 Predictive cohort study

#### 2.2.1 Patients

In this predictive cohort study, we enrolled 120 non-dialysis patients aged 18–75 yearsbetween December 2016 and January 2022. This cohort study was comprised of subjects from Dongzhimen Hospital, Beijing University of Chinese Medicine. The exclusion criteria were identical tothose used in the cross-sectional study. The criteria for stage IV DKD were as follows: (1) UAER>300 mg/24 h or UACR>300 mg/g; (2) 24h UTP>0.5g, and (3) eGFR≥15 mL/min/1.73 m^2^.

#### 2.2.2 Data collection

Patients were followed up at three-month intervals until the end of the study period or the occurrence of primary endpoint events. The collected data were the same as those used in the cross-sectional study. The primary outcome was disease progression, which was defined as progression to ESRD (sustained eGFR<15mL/min/1.73 m^2^), having received renal replacement, or death from a variety of causes associated with kidney diseases.

#### 2.2.3 Statistical analysis

The prognostic value of these variables was further tested using univariate and multivariate Cox proportional hazards regression analyses. Kaplan-Meier survival curves were plotted and differences in survival between groups were estimated using the log-rank test. To calculate the ORs for DKD according to the quartiles of SLPI levels, and a trend test was conducted. Statistical significance was set at p ≤ 0.05. Kaplan-Meier analysis was performed using GraphPad Prism 8.0.

## 3 Results

### 3.1 Cross-sectional study

#### 3.1.1 Baseline characteristics

A total of 266 patients with DKD were enrolled in the multi-center cross-sectional study, including 175 males and 91 females with a mean age of 57.94 ± 8.60 years. Statistically significant differences were observed in age, male-to-female ratio, duration of diabetes, and duration of proteinuria among the three groups (p<0.01). Statistically significant differences were also observed in the levels of SBP, HGB, urea, Scr, UA, eGFR, 24h UTP, Alb, CHO, TG, and LDL (**Table 1**). Trends in clinical laboratory parameters were in accordance with the course of DKD.

**Table 1:** Baseline characteristics of the study population in different stages of DKD.

	Early	Established	Advanced	*P*
Cases(Male/Female)	92 (43/49)	92 (74/18)	82 (58/24)	<0.001
Age(years)	59.43 ± 8.42	55.07 ± 8.98	59.48 ± 7.58	<0.001
Duration of diabetes(years)	12.50 ± 10.00	15.00 ± 8.75	17.00 ± 10.00^A^	0.006
Duration of proteinuria (months)	12.00 ± 23.00	24.00 ± 52.75	36.00 ± 48.50	<0.001
SBP(mm Hg)	130.00 ± 15.50	136.00 ± 10.00	136.00 ± 16.75	<0.001
DBP(mm Hg)	79.00 ± 10.00	80.00 ± 9.00	78.00 ± 15.00	0.077
BMI(kg/m²)	25.10 ± 6.18	25.27 ± 3.54	25.59 ± 4.94	0.456
HGB(g/dl)	13.75 ± 3.05	13.30 ± 2.75	11.60 ± 2.32	<0.001
GLU(mmol/L)	8.04 ± 3.51	8.47 ± 4.28	7.27 ± 4.12	0.087
HbA1c(%)	6.90 ± 1.60	6.97 ± 1.58	6.80 ± 1.40	0.462
Urea(mmol/L)	5.46 ± 1.68	6.81 ± 3.51	11.25 ± 4.66	<0.001
Scr(μmol/L)	60.30 ± 23.47	80.50 ± 30.85	150.00 ± 79.70	<0.001
UA(μmol/L)	326.60 ± 123.80	378.55 ± 102.60	403.50 ± 131.65	<0.001
eGFR(mL/min/1.73m^2^)	121.86 ± 55.23	88.51 ± 48.81	40.59 ± 18.51	<0.001
24h UTP(mg/24h)	220.84 ± 200.50	1583.00 ± 2499.75	3448.00 ± 3517.25	<0.001
Alb(g/L)	43.16 ± 6.88	40.1 ± 7.83	36.50 ± 8.53	<0.001
CHO(mmol/L)	4.42 ± 1.38	4.96 ± 2.02	4.76 ± 2.30	0.001
TG(mmol/L)	1.66 ± 1.10	1.82 ± 1.49	1.92 ± 1.87	0.004
LDL(mmol/L)	2.53 ± 1.09	2.87 ± 1.16	2.72 ± 1.26	0.045

SBP, systolic blood pressure; DBP, diastolic blood pressure; BMI, body mass index; HGB, haemoglobin; GLU, Glucose; HbA1c, hemoglobin A1c; eGFR, estimated glomerular filtration rate; Scr, serum creatinine; UA, uric acid; 24h UTP, 24 hours urinary total protein; Alb, albumin; CHO, serum cholesterol; TG, triglyceride; LDL, low-density lipoprotein.

#### 3.1.2 SLPI and decline of kidney function

##### 3.1.2.1 Serum SLPI levels at different stages of DKD

**Figure 1:**
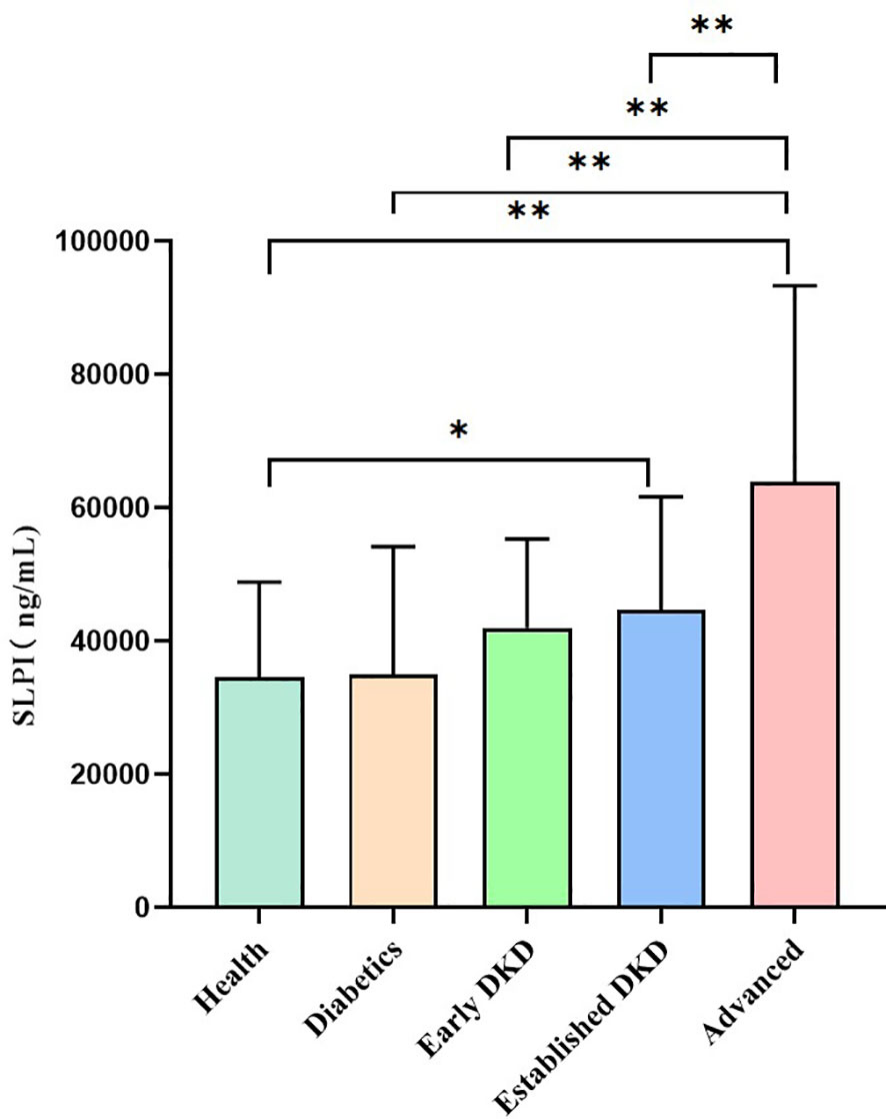
Serum SLPI levels among groups. Notes: Healthy group versus other groups (*p<0.05); healthy group versus other groups (**p<0.01); diabetes group versus other groups (**p<0.01); early group versus other groups (**p<0.01); established group versus other groups (**p<0.01).

As shown in **Figure 1**, serum SLPI levels increased among healthy controls, the diabetes group, and the early, established and advanced stages of DKD, with statistically significant differences observed among the five groups (H = 84.26, p<0.01). In the comparison between the two groups, the serum SLPI levels were significantly higher in the established group and advanced group than in the healthy controls (p<0.05, p<0.01, respectively); however, no statistically significant difference was observed in the SLPI levels between the diabetes group and early group compared to the healthy group. Additionally, patients in the advanced group has significantly higher SLPI levels than those in the other groups (p<0.01).

##### 3.1.2.2 Correlation between serum SLPI levels and routine clinical parameters in patients with DKD

We evaluated the correlation between serum SLPI levels and baseline parameters in patients with DKD. As shown in **Table 2**, the serum SLPI levels were negatively correlated with eGFR (r= -0.564, p<0.01) and positively correlated with urea (r=0.532, p<0.01) and Scr (r=0.604, p<0.01). Moreover, serum SLPI levels were positively correlated with 24h UTP (r = 0.372, p<0.01) and the UA levels (r = 0.233, p<0.01). In addition, serum SLPI levels were negatively correlated with the HGB (r = -0.158, p<0.01) and Alb (r = -0.126, p<0.05) levels, whereas they were positively correlated with SBP (r = 0.161, p<0.01) and the duration of proteinuria(r = 0.233, p<0.01). These results suggest that the serum SLPI levels in patients with DKD are positively correlated with disease progression.

**Table 2:** Correlation between serum SLPI and clinical parameters.

Parameters	Serum SLPI
	Correlation coefficients(*r*_s_)
eGFR(mL/min/1.73m^2^)	-0.564^**^
24h UTP(mg/24h)	0.372^**^
Urea(mmol/L)	0.532^**^
Scr(μmol/L)	0.604^**^
UA(μmol/L)	0.233^**^
HbA1c(%)	-0.132^*^
Alb(g/L)	-0.126^*^
CHO(mmol/L)	0.135^*^
HGB(g/dl)	-0.158^**^
SBP(mmHg)	0.161^**^
Duration of proteinuria(months)	0.233^**^

^*^P<0.05; ^**^P<0.01.

##### 3.1.2.3 Evaluation of the role of SLPI level in DKD diagnosis by ROC curve analysis

**Figure 2:**
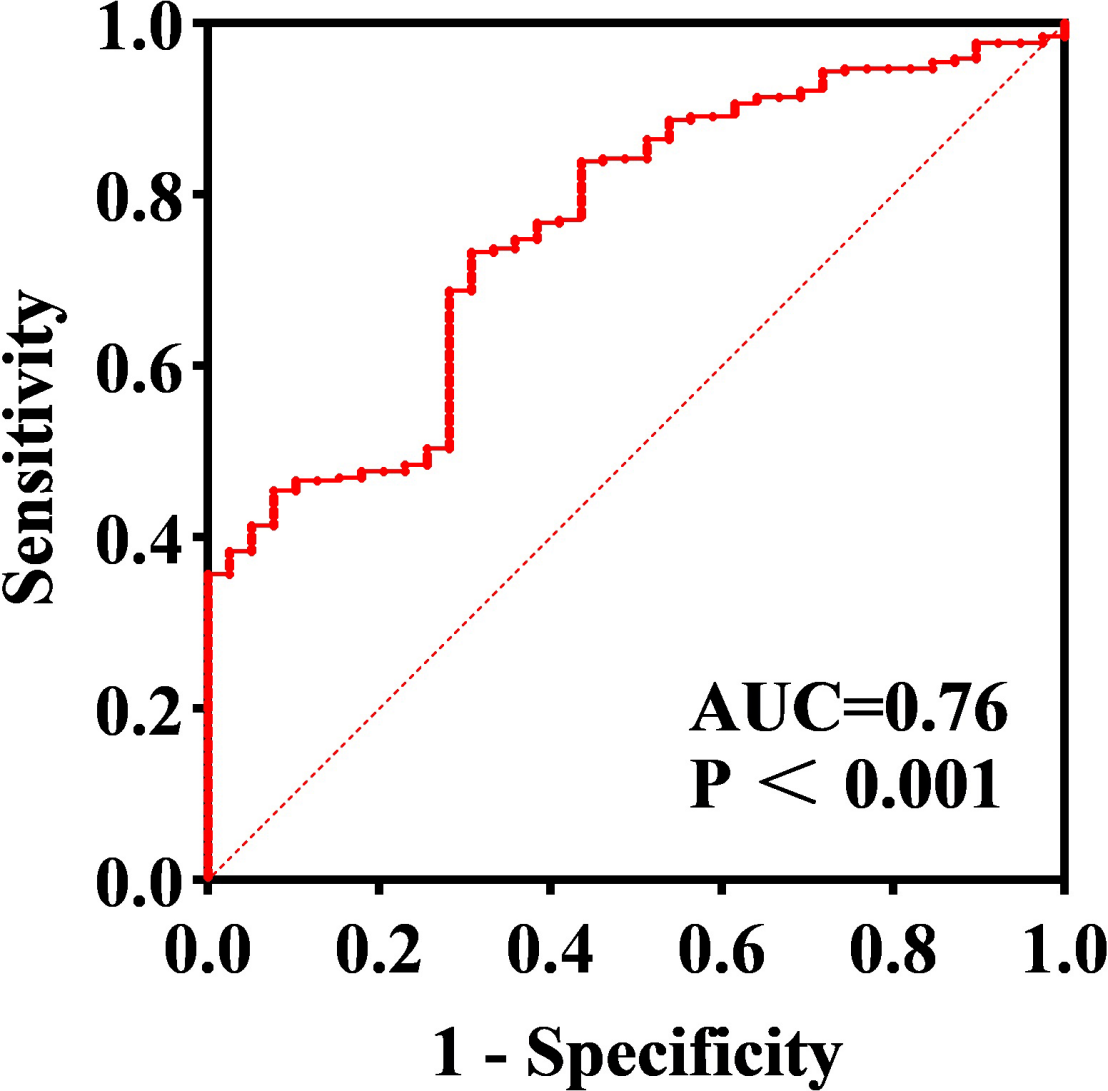
ROC curve of serum SLPI for predicting DKD. Cut-off SLPI level of 39.11 ng/ml offered optimal differentiation between patients with and without DKD (AUC=0.76, 95% CI=[0.69–0.83]).

All subjects were grouped into DKD and non-DKD groups, and the role of serum SLPI level as a predictor was tested using ROC curve analysis. ROC analysis showed that an SLPI cutoff level of 39.11ng/ml offered optimal differentiation between patients with and without DKD (73.31% sensitivity and 69.23% specificity). The ROC analysis indicated that the serum SLPI level tended to offer diagnostic value for DKD (AUC=0.76, 95% CI [0.69, 0.83], p<0.01) (**Figure 2**). Therefore, the results revealed that serum SLPI levels may be a factor in the diagnosis of DKD.

### 3.2 Predictive cohort study

#### 3.2.1 Comparison of participant data in low and high SLPI expression groups

A total of 120 patients with stage IV DKD were included in the predictive cohort study. Patients with DKD were divided into low and high SLPI expression groups based on the median serum SLPI level (51.61 ng/mL). **Table 3** shows the clinical and laboratory characteristics of the study population. No significant differences were observed betweenthe groups in term of age, sex, duration of diabetes, or duration of proteinuria. However, patients with higher SLPI levels had a lower eGFR and a higher frequency of renal endpoint eventsthan patients with lower SLPI levels (p<0.01).

**Table 3:** Baseline characteristics of participants in the low and high SLPI expression groups.

Characteristics	Low SLPI expression group(*n*=60)	High SLPI expression group(*n* =60)	Statistics	*P*
Age (years)	57.28 ± 8.17	56.25 ± 8.8	*T* = 0.67	0.51
Male, *n* (%)	42(70.00)	48(80.00)	*χ²* = 1.60	0.21
Duration of diabetes (years)	14.82 ± 6.08	16.4 ± 7.09	*t* = -1.32	0.19
Duration of Proteinuria (months)	34.02 ± 36.24	45.65 ± 52.14	*t* = -1.42	0.16
24hUTP (mg/24h)	3195.62 ± 3443.50	3591.41 ± 3187.63	*Z* = -1.74	0.081
eGFR (mL/min/1.73m^2^)	87.77 ± 54.57	54.80 ± 28.99	*Z* = -4.91	<0.01
HbA1c (%)	7.03 ± 1.08	6.61 ± 1.03	*t* = 2.204	0.029
Renal endpoint events, n (%)	14(23.33)	32(53.33)**	*χ²=11.42*	<0.01

^*^P<0.05; ^**^P<0.01.

#### 3.2.2 Prognostic value of serum SLPI level on renal endpoint events

**Figure 3:**
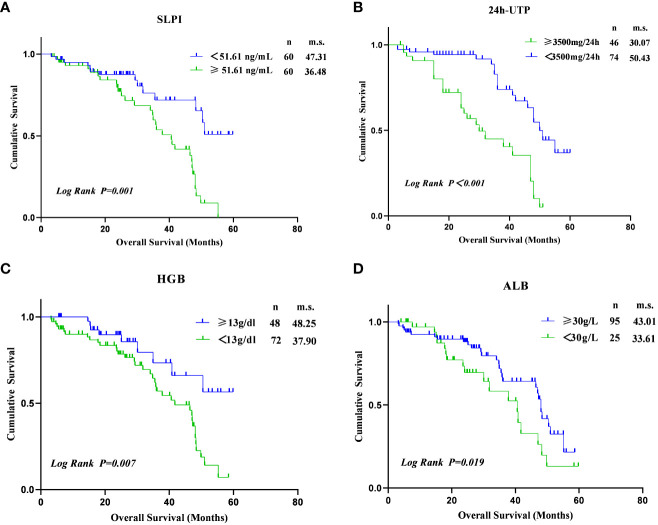
Kaplan–Meier estimates of survival during follow-up in DKD. **(A)** Between SLPI≥51.61 ng/mL and SLPI<51.61ng/ml (log-rank p=0.001). **(B)** Between 24h UTP≥3500mg/24h and 24h UTP<3500mg/24h (log-rank p<0.001). **(C)** Between HGB<13g/dl and HGB≥13g/dl (log-rank p=0.007); **(D)** Between ALB<30g/L and ALB≥30g/L (log-rank p=0.019).

The mean follow-up duration for the cohort study was 2.32 ± 1.30 years. As shown in **Figure 3**, elevated SLPI levels (≥51.61 ng/mL) were associated with an increased risk of renal endpoint events (log-rank p=0.001). Furthermore, an increased risk of developing renal endpoint events was observed in patients with 24h UTP≥3.5g (log-rank p<0.01). In addition, a decrease in HGB levels (<13g/dl) was associated with a higher risk of developing renal endpoints (log-rank p=0.007), as well as reduced ALB levels(< 30g/L) (log-rank p=0.019). Therefore, Kaplan-Meier analysis showed a significantly faster progression to renal clinical endpoint events in subjects with SLPI ≥51.61 ng/mL, 24 h UTP≥3.5 g, HGB <13 g/dl, and ALB <30 g/L.

Next, we performed univariate and multivariate analyses to identify risk factors associated with renal endpoint events in patients with DKD. In the univariate analysis, variables associated with renal endpoint events were high expression SLPI level (HR= 2.95, p=0.001), 24h UTP≥3500mg (HR = 3.85, p<0.001), HGB<13g (HR = 2.65, p=0.010), Alb<30g (HR = 2.19, p=0.022) and urea≥7.1 mmol/L (HR = 9.59, p=0.002). Risk factors for renal clinical endpoint events were further analyzed using a multivariate regression model to determine the most important factors. Based on the multivariate regression model, only serum SLPI levels ≥ 51.61 ng/mL (HR = 2.95, 95% CI[1.55, 5.60], p=0.001), 24h UTP≥3.5g (HR = 3.02, 95% CI[1.66, 5.52], p<0.001), HGB<13g (HR = 3.18, 95% CI[1.49, 6.80], p=0.003), Alb<30g (HR = 2.19, 95% CI[1.12, 4.28], p=0.021) and urea≥7.1 mmol/L (HR = 8.27, 95% CI[1.96, 34.93], p=0.004) were independently associated with renal endpoint events in DKD patients. Survival prediction analysis indicated that higher SLPI levels, massive proteinuria, increased urea levels, and lower ALB and HGB levels increased the probability of renal endpoint events in patients with DKD (**Table 4**).

**Table 4:** Cox regression analysis for outcomes in DKD.

Factors	Univariable		Multivariable model ^a^	
	*HR*(95% *CI*)	*P value*	*HR*(95% *CI*)	*P value*
Sex				
male	1.0		1.0	
female	0.83(0.41,1.69)	0.614	0.57(0.27,1.17)	0.124
Age(years)				
<65	1.0		1.0	
≥65	1.15(0.74,2.92)	0.270	1.51(0.76,2.99)	0.243
Serum SLPI(ng/mL)				
low expression group	1.0		1.0	
high expression group	2.95(1.55, 5.62)	0.001	2.95(1.55, 5.60)	0.001
24h UTP(mg/24h)				
<3500mg	1.0		1.0	
≥3500mg	3.85(2.09, 7.06)	<0.001	3.02(1.66, 5.52)	<0.001
HGB(g/dl)				
≥13g	1.0		1.0	
<13g	2.65(1.27,5.53)	0.010	3.18(1.49, 6.80)	0.003
Alb(g/L)				
≥30g	1.0		1.0	
<30g	2.19(1.12,4.29)	0.022	2.19(1.12, 4.28)	0.021
Urea(mmol/L)				
<7.1	1.0		1.0	
≥7.1	9.59(2.29, 40.26)	0.002	8.27(1.96, 34.93)	0.004

a Each risk factor (age, serum SLPI, 24h UTP, HGB, Alb, Urea) has been adjusted for the other factors.

In addition, we further grouped SLPI into quartiles to observe trends in disease progression in thedifferent SLPI subgroups, as well as to analyze the relationship between SLPI levels and disease progression in DKD. As shown in **Table 5**, the disease progression rate of DKD progressively increased from 16.7% (5/30) to 53.3% (16/30) across the SLPI quartiles. Similar trends were observed in each model. With an increase in the SLPI levels, the incidence of endpoint events in DKD patients also increased (all p<0.05).

**Table 5:** Odds ratios and 95% confidence interval for DKD and its individual components according to quartile of SLPI.

	Quartile of SLPI				*P* Value for Trend
	Q1	Q2	Q3	Q4	
	≤40.93	40.94-51.61	51.62-67.32	>67.32	
Patients, n	30	31	29	30	
Disease Progression, n	5	10	15	16	0.001
Model 1	1.00	3.361(1.141-9.899)	5.236(1.840-14.897)	5.397(1.965-14.822)	0.012
*P* Values		0.028	0.002	0.001	
Model 2	1.00	3.338(1.134-9.826)	5.344(1.850-15.434)	5.724(2.071-15.821)	0.027
*P* Values		0.029	0.002	0.001	
Model 3	1.00	4.180(1.283-13.622)	4.843(1.502-15.616)	4.815(1.510-15.360)	0.020
*P* Values		0.018	0.008	0.008	

Model 1: crude, no adjustment; Model 2: adjusting for age, gender, duration of diabetes, and duration of proteinuria; Model 3: adjusting for age, gender, duration of diabetes, duration of proteinuria, Urea, GLU, HbA1c, UA, and ALB.

## 4 Discussion

**Figure 4:**
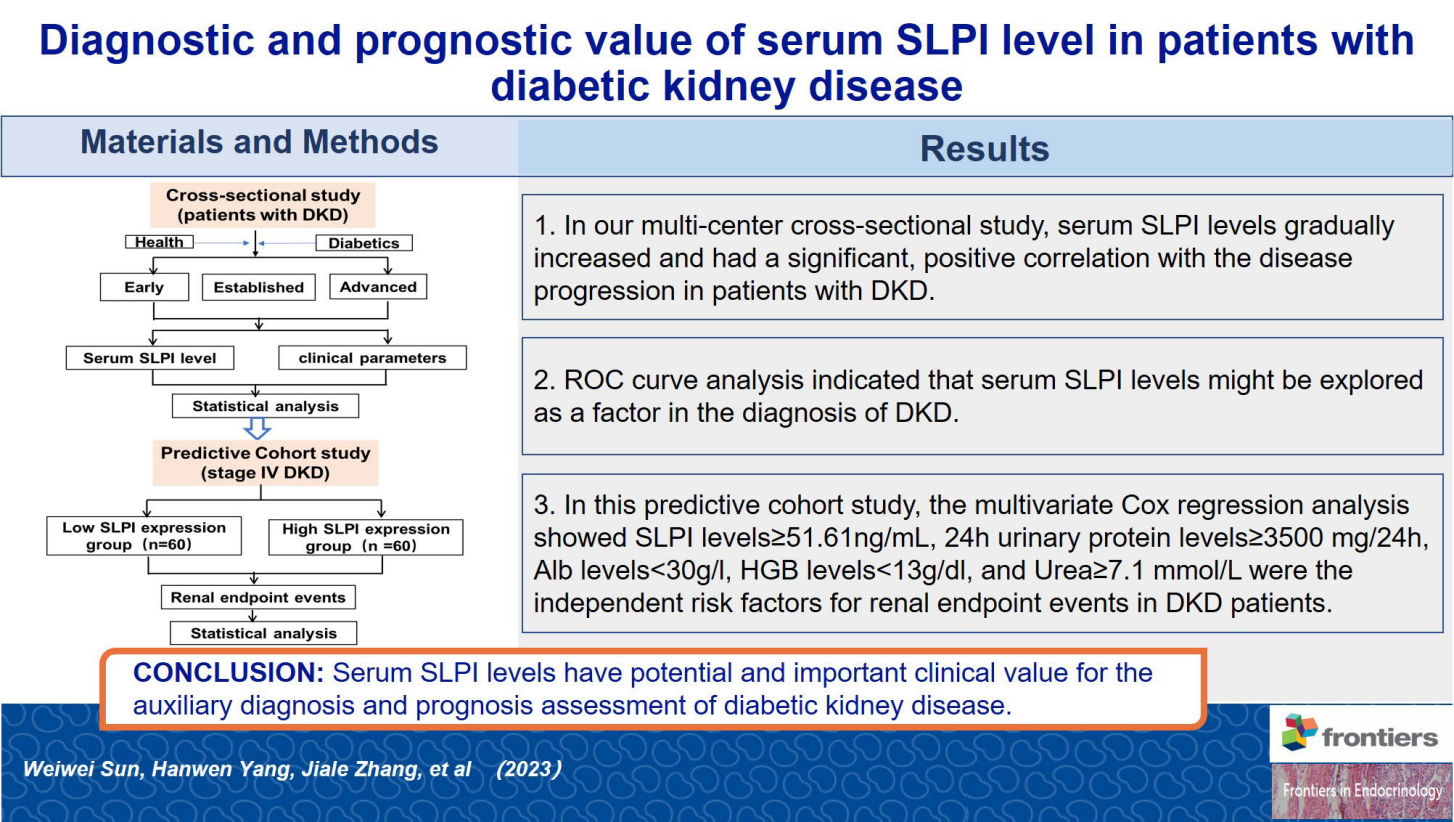
Graphical abstract.

The discovery of novel biomarkers is extremely important for the early diagnosis and prediction of adverse outcomes of DKD. As shown in **Figure 4** this cross-sectional study evaluated the diagnostic and prognostic value of serum SLPI levels in patients with DKD. Serum SLPI levels were found to gradually increase during the occurrence and progression of DKD. Furthermore, serum SLPI levels in patients with DKD were positively correlated with aggravation of the disease, and act as an important reference value for the diagnosis of DKD. In this predictive cohort study, we further observed that elevated SLPI levels, 24 h UTP≥3.5 g, HGB <13 g/dl, and ALB < 30 g/L were independent risk factors and increased the probability of renal endpoint events in patients with DKD. In addition, we further divided the SLPI into quartiles, observed trends in disease progression across different SLPI subgroups, and analyzed the relationship between the SLPI and DKD progression. The results showed that an increase in the SLPI level was a risk factor for DKD progression. After adjusting for a range of relevant factors, SLPI remained an independent risk factor for DKD progression. These results highlighted the diagnostic and prognostic potential of serum SLPI levels in DKD.

We investigated the association between serum SLPI levels and renal function at different stages of DKD in a large multi-center cross-sectional study. For these 266 patients, the results showed that serum SLPI was expressed at higher levels in the early-to-moderate stages of DKD and gradually increased with the progression of DKD, suggesting that SLPI plays a role in the initiation and progression of DKD. Moreover, SLPI levels were highly correlated with renal dysfunction in patients with DKD. This validates the previous observation of a strong correlation between the SLPI and DKD in a smaller cross-sectional study (18). Circulating SLPI levels consistently increase with the progression of metabolic dysfunction and are independently associated with metabolic and inflammatory markers in a prospective cross-sectional study (17, 19). This result is consistent with the increased expression of SLPI in DKD, a well-known chronic inflammatory state.

We also found that elevated SLPI levels were positively correlated with renal dysfunction in patients with DKD. This suggests that SLPI, as an alarm reactant, increases upon stimulation with renal injury and reflects the severity of the inflammatory situation during DKD. The mechanism of SLPI expression level in the initiation and progression of DKD may be related to counterbalance the pro-inflammatory condition that increases the risk for DKD by attenuating neutrophil recruitment and phagocytosis, inhibiting the monocyte/macrophage response to endotoxins and suppressing the activation of transcription factor NF-κB (20, 21). Additionally, a positive correlation was observed between SLPI and hyperglycemia, hypertension, and hyperlipidemia, which are common risk factors for DKD. Given the increased chronic stress and inflammation in DKD, it is tempting to speculate that the upregulation of circulating SLPI may be associated with metabolic disorders to meet the demands of anti-stress and anti-inflammatory activities (22, 23).

We analyzed the diagnostic value of the serum SLPI in patients with DKD using ROC curves. The results showed that serum SLPI levels had moderate diagnostic value for DKD, which validated the diagnostic potential of SLPI as a biomarker, previously identified in a large cross-sectional study (18). Similarly, a prospective observational study implicated SLPI in the pathogenesis of kidney diseases and identified it as a postoperative biomarker for the early diagnosis of acute kidney disease (15). This may be considered one of the best predictors of DKD progression. However, given that SLPI may widely interfere with kidney injury, the measurement of serum SLPI elevation alone is not sufficient to diagnose DKD. Further studies are needed to compare SLPI levels between patients with and without DKD. Current clinical methods used to predict the initiation and progression of DKD are usually an elevated urinary albumin-to-creatinine ratio (UACR) or microalbuminuria of 30~300 mg/24h (24). However, high interindividual variability in the mild-to-moderate stages of DKD is a major limitation for accurate early diagnosis and prognosis (25, 26). In addition, other biomarkers reported in recent studies, including renal injury(such as KIM-1, β2-MG, Cys-C, and YKL-40) and proinflammatory factors (such as TNF receptor-1/2, suPAR, and MCP-1) showed a relatively lower sensitivity and specificity for predicting kidney injury associated with diabetes (4, 5, 27). Thus, it is more feasible to establish routine diagnostic biomarkers for kidney injury in DKD. This study revealed that its combination with microalbuminuria and other biomarkers may be more accurate for the early diagnosis and prognosis of DKD. Moreover, higher serum SLPI levels may be used to identify individuals with diabetes who are at a risk of eGFR decline.

At present, the prognostic value of SLPI in DKD has not yet been evaluated in detail. In our cohort study, patients with stage IV DKD were selected as the study subjects because of their relatively rapid rate of progression and because it is easy to observe renal clinical endpoint events. We found that a higher serum SLPI concentration was independently associated with an elevated risk of renal endpoint events among individuals with stage IV DKD, even after adjusting for clinical risk factors. To date, because of SLPI’s ability to protect against tissue repair, studies investigating SLPI and kidney disease in humans have mostly focused on acute kidney injury (15, 16). To the best of our knowledge, The present study is the first to describe the association between elevated serum SLPI concentrations and adverse renal outcomes in patients with DKD. These results are consistent with previous findings that high SLPI expression is associated with poor prognosis in cancers (28–30). However, the cause-and-effect relationship between increased SLPI expression and disease progression has not yet been studied, and most studies have indicated that the upregulation of SLPI is associated with proinflammatory process. In addition, whether elevated serum SLPI levels lead to renal damage by limiting the organism’s ability to counter overactivated inflammatory and metabolic stress during DKD has not yet been ruled out (31, 32). Thus, additional studies on the exact biological contribution of serum SLPI to DKD progression are required. Additionally, we also found that DKD patients with 24h UTP≥3.5g, HGB < 13g/dl, and ALB < 30g/L showed a significantly faster progression to renal clinical endpoints, which were consistent with previous studies in DKD patients. Albuminuria is an established risk factor of kidney failure. Previous studies have indicated that albuminuria is a risk factor for renal prognosis in patients with type 2 diabetes (33). In a previous prospective, multicenter cohort study of 1138 pre-dialysis CKD, decreased serum albumin and HGB were identified as risk factors for renal endpoints (34). Moreover, measurements of urea levels revealed a valuable prognostic capacity to predict progression to renal endpoint events. Taken together, these results indicated that SLPI levels≥51.61ng/ml in combination with 24h UTP≥ 3.5g, HGB < 13 g/dl, ALB < 30 g/L and urea ≥ 7.1mmol/L may be the optimal thresholds for predicting and identifying high-risk populations.

The mechanisms underlying the protective role of SLPI in this process are diverse. DKD is a chronic inflammatory disease, triggered by metabolic disturbances, hemodynamic changes, increased oxidative stress, and profibrotic mediators (35). SLPI has been identified as a potent inflammation-suppressing protein that is triggered in proportion to the degree of inflammation, playing a role in suppressing the inflammatory environment. Higher expression of SLPI can antagonize the activation of the inflammatory transcription factor NF-κB and counteract the effect of proinflammatory cytokines, thus alleviating kidney damage and promoting proximal tubular cell regeneration in a diabetic state (23). Moreover, SLPI is recognized as a molecule that favors the host via its immunomodulatory, anti-proteolytic, and anti-microbial properties (6). SLPI regulates the pro-immunogenic function of neutrophil extracellular traps and inhibits the activity of several proteolytic enzymes, making it a component of the defense mechanisms in the kidney (22). However, to date, the specific role of SLPI in DKD pathogenesis remains unclear and requires further examination in experimental studies.

To the best of our knowledge, this study is the first to comprehensively evaluate the potential clinical value of serum SLPI levels for the combined diagnosis and prognosis of DKD. However, several limitations and potential drawbacks of this study should beconsidered. First, the sample sizes of both the cross-sectional and predictive cohort studies were relatively small. Furthermore, the study’s limitations to Beijing hospitals may limit the applicability of the findings to a larger population. Among these, the external validity of the study could be improved by replicating it with a nationwide multi-center clinical research with a larger sample size. Second, wewere unable to directly establish a causal relationship between elevated SLPI levels and DKD progression. Whether an elevated SLPI level is the cause of the occurrence and progression of DKD or an accompanying factor of DKD will be further verified through subsequent large-sample clinical trials and precise experimental studies. in addition, the segment of the study that examined future possibilities had a limited timeframe, potentially not capturing a comprehensive picture over an extended period. In future studies, we will increase the follow-up period, which may provide a deeper understanding of the long-term predictive usefulness of the SLPI. Another limitationis that we only observed patients with stage IV DKD in this predictive cohort study, which could not provide the risk of clinical renal endpoints for patients with other stages of DKD. Furthermore, the exclusion criteria, targeting patients with specific conditions, may introduce selection bias, limiting the applicability of the findings to a broader DKD patient population with diverse comorbidities. The prognostic utility of SLPI levels in other study populations is warranted to further investigate under the setting of DKD. Third, potential confounding factors, such as medications that may affect SLPI levels and the course of DKD, were not sufficiently addressed in this study. Therefore, we will further explore the available data on the effect of medications on SLPI levels during the course of DKD to provide a comprehensive analysis. Moreover, the absence of information on the ethnic or racial composition of the study population is notable because such differences can influence disease progression and biomarker performance. Thus, larger studies using genetic data, serum SLPI levels, and a greater number of DKD events are required to elucidate whether genetically mediated SLPI levels are associated with DKD.

In conclusion, we demonstrated that the measurement of serum SLPI levels had auxiliary diagnostic and prognostic value in patients with DKD. In future studies, we will expand the sample size to verify its predictive value in the diagnosis and prognosis of DKD and explore the specific functional properties of SLPI in DKD through animal and cell experiments.

## Data availability statement

The raw data supporting the conclusions of this article will be made available by the authors, without undue reservation.

## Ethics statement

The studies involving humans were approved by the Medical Ethics Committee of Dongzhimen Hospital Affiliated to Beijing University of Chinese Medicine (DZMEC-KY-2016-95). The studies were conducted in accordance with the local legislation and institutional requirements. The participants provided their written informed consent to participate in this study.

## Author contributions

WS: Investigation, Methodology, Project administration, Writing – original draft. HY: Investigation, Methodology, Writing – original draft. JZ: Data curation, Investigation, Software, Writing – review & editing. SW: Data curation, Investigation, Writing – review & editing. QW: Investigation, Writing – review & editing. CC: Data curation, Investigation, Writing – review & editing. JY: Data curation, Investigation, Writing – review & editing. ZC: Data curation, Formal analysis, Project administration, Writing – review & editing. HZ: Data curation, Project administration, Supervision, Writing – review & editing. YW: Conceptualization, Funding acquisition, Project administration, Supervision, Writing – review & editing.
